# Packaged Droplet Microresonator for Thermal Sensing with High Sensitivity

**DOI:** 10.3390/s18113881

**Published:** 2018-11-11

**Authors:** Xiaogang Chen, Liang Fu, Qijing Lu, Xiang Wu, Shusen Xie

**Affiliations:** 1 Key Laboratory of Optoelectronic Science and Technology for Medicine of Ministry of Education, Provincial Key Laboratory for Photonics Technology, Fujian Normal University, Fuzhou 350007, China; xgchen01@139.com (X.C.); fuliang1995@126.com (L.F.); ssxie@fjnu.edu.cn (S.X.); 2 Department of Optical Science and Engineering, Fudan University, Shanghai 200433, China; wuxiang@fudan.edu.cn

**Keywords:** whispering gallery mode, package droplet, quasi-droplet, thermal sensing

## Abstract

Liquid droplet and quasi-droplet whispering gallery mode (WGM) microcavities have been widely studied recently for the enhanced spatial overlap between the liquid and WGM field, especially in sensing applications. However, the fragile cavity structure and the evaporation of liquid limit its practical applications. Here, stable, packaged, quasi-droplet and droplet microcavities are proposed and fabricated for thermal sensing with high sensitivity. The sensitivity and electromagnetic field intensity distribution are analyzed by Mie theory, and a quantified definition of the quasi-droplet is presented for the first time to the best of our knowledge. By doping dye material directly into the liquid, lasing packaged droplet and quasi-droplet microcavity sensors with a high thermal sensitivity of up to 205.3 pm/°C are experimentally demonstrated. The high sensitivity, facile fabrication, and mechanically robust properties of the optofluidic, packaged droplet microresonator make it a promising candidate for future integrated photonic devices.

## 1. Introduction

Recently, microcavity based on the whispering gallery mode (WGM) has been demonstrated with extremely high quality (*Q*) factors and ultra-small mode volumes [1,2]. Because WGM resonators can concentrate electromagnetic energy in the small space by a process of continuous total internal reflection, the intracavity power density is greatly enhanced. The WGM microcavity has been widely used in various applications, such as low-threshold microlasers [3,4], nonlinear optical effects [5,6], integrated photonic devices [7,8], and sensors [9,10,11,12]. Several types of the WGM resonators have been proposed to date, such as microsphere [13], microdisk [14], microbubble/microbottle [15], and microring [16]. The resonant wavelengths of WGM microcavity are highly influenced by external factors, e.g., temperature [17] and refractive index [18]. This leads to ultrahigh sensitivity in sensing applications. Traditionally, the WGM sensors employ solid-state microcavity. However, the low thermal expansion coefficient and thermo-optical coefficient (TOC) of solid material limited its applications in thermal sensors.

Liquid droplets as a unique type of optical resonator have first been studied in 1970s and been researched extensively since then [19,20]. The liquid resonators, which have extremely high *Q* factors resulting from the smooth surface and spherical shape, are suitable for various sensing applications [21,22,23]. Due to the large TOC, the liquid resonators exhibit a high resolution for thermal sensing. A mounting number of designs of liquid resonators was proposed and developed for different applications in recent years [24,25,26]. However, there are several disadvantages which prevent the practical utilization of liquid droplets. For example, the instability of the droplet structure makes it difficult to manipulate. The short lifetime of the liquid droplet due to evaporation also limits its practical applications.

Many researchers have attempted to resolve these aforementioned limitations. One approach is to use two immiscible liquids. One microdroplet is immersed into another liquid medium by a self-assembled method [27,28,29]. This design can prevent the microdroplet from evaporating and protect it from external contaminants. However, this approach requires a complex experimental setup and is difficult to apply in a small space [28]. The other approach is the development of the quasi-droplet microcavity [4,30,31]. Lee et al. [4] fabricated a quasi-droplet optofluidic ring resonator (OFRR) laser based on a fused silica microbubble with an extremely thin wall. A wall thickness as thin as 560 nm was achieved. Ward et al. [31] presented experimental results on the detection of 100 nm and 500 nm polystyrene particles in aqueous solution using thin-walled (780 nm), hollow WGM supporting quasi-droplet modes. Due to the ultrathin wall thickness, the quasi-droplet modes exhibit high sensitivity. However, the microbubble with thin wall is fragile and difficult to fabricate.

In this study, we proposed and demonstrated a novel and high-sensitivity thermal sensor based on a quasi-droplet WGM microbubble resonator (MBR) with a thick wall and a packaged droplet resonator. The microbubble for the quasi-droplet did not require an extremely thin wall and special handling. Firstly, a numerical simulation by Mie theory was studied to determine experimental rationality. A quantified definition of the quasi-droplet with a thick wall was presented for the first time to the best of our knowledge. The lasing, packaged quasi-droplet microcavity sensor with a high thermal sensitivity was experimentally demonstrated. For further study, the packaged droplet resonator was fabricated by a hydrofluoric etching process. The ultimate sensibility in a packaged droplet thermal sensor was thus realized. In addition, the packaged microdroplet laser was pumped and its spectrum was collected through free optics, which eliminates the need for phase-matched prism or tapered micro/nanofiber. The proposed on-chip microdroplet resonator with a packaged structure has the advantage of simple, compact, and robust characteristics, which will have significant potential in regard to future integrated photonic devices.

## 2. Theoretical Analysis

**Figure 1:**
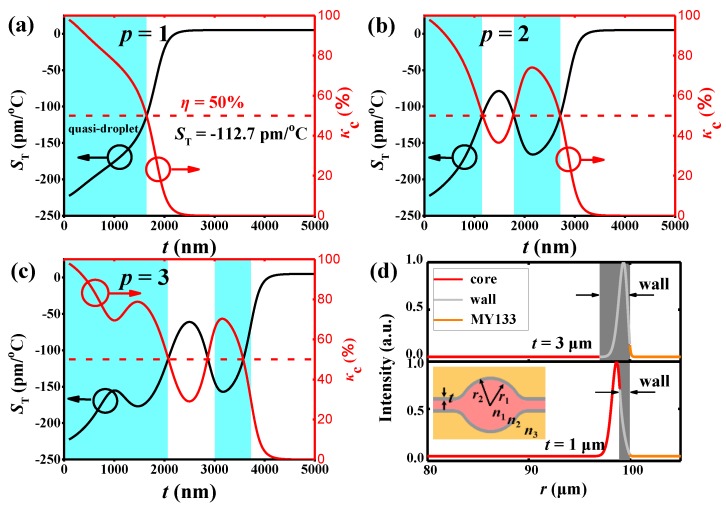
(**a**–**c**) The calculated sensitivity (left Y axis) and the normalized percentage of electromagnetic energy in the core (right Y axis) for the *p* = 1–3 modes as a dependence of the thickness of the microbubble wall. The red broken line is a value of 50%. The cyan shadow shows the regime of the quasi-droplet. (**d**) The electromagnetic field distribution of *p* = 1 mode with wall thicknesses of 1 μm and 3 μm (gray shadow), respectively. Inset: the three-layer Mie model. Here: *r*_2_ = 100 μm, *n*_1_ = 1.479, *n*_2_ = 1.45, *n*_3_ = 1.33.

For a deeper understanding of the electromagnetic field distribution and the sensitivity dependent on the thickness of the microbubble wall, the three-layer Mie model was used in the simulation and illustrated in Figure 1d. The radial distribution of the WGM of the microbubble can be written as [32]:

(1) $$E_{m,p}(r)=\{\begin{array}{c} AJ_{m}(k_{0}^{\left(p\right)}n_{1}r)\\ BJ_{m}(k_{0}^{\left(p\right)}n_{2}r)+CH_{m}^{\left(p\right)}(k_{0}^{\left(p\right)}n_{2}r)\\ DH_{m}^{\left(p\right)}(k_{0}^{\left(p\right)}n_{3}r) \end{array}\begin{array}{c} r\leq r_{1}\\ r_{1}\leq r\leq r_{2}\\ r_{2}\leq r \end{array},$$

where *J_m_* and $H_{m}^{\left(p\right)}$ are the *m*th Bessel function and the *m*th Hankel function of the first kind, respectively. *p* and *m* are the radial number and azimuthal number, respectively. The refractive indices of the core, wall, and the surrounding medium are denoted by *n*_1_, *n*_2_, and *n*_3_. The terms *r*_1_ and *r*_2_ represent the inner and outer radius of the microbubble, respectively, and $k_{0}^{\left(p\right)}$ is the amplitude of the wave vector in a vacuum for the *p*-order radial WGM.

The sensitivity for the temperature change, *S_T_*, is related to the shift of the WGM resonator wavelength (Δ*λ*) and expressed as [33]:

(2) $$S_{T}=\frac{\partial \lambda }{\partial T}=\kappa_{c}\frac{\partial \lambda }{\partial n_{1}}\frac{\partial n_{1}}{\partial T}+\kappa_{s}\frac{\partial \lambda }{\partial n_{2}}\frac{\partial n_{2}}{\partial T}+\kappa_{b}\frac{\partial \lambda }{\partial n_{3}}\frac{\partial n_{3}}{\partial T}=\kappa_{c}\frac{\lambda }{n_{1}}\alpha_{1}+\kappa_{s}\frac{\lambda }{n_{2}}\alpha_{2}+\kappa_{b}\frac{\lambda }{n_{3}}\alpha_{3},$$

where *κ_c_*, *κ*_s_, and *κ_b_* are the proportion of electromagnetic field in the core, shell, and surrounding medium, respectively, while *n*_1_, *n*_2_, and *n*_3_ are their refractive indices. *α*_1_ = *∂n*_1_/*∂T*, *α*_2_, and *α*_3_ are the TOC of the core, shell, and surrounding medium respectively.

Figure 1a–c shows the normalized *κ_c_* (red line) and *S_T_* (black line) for the first three radial modes (*p* = 1–3) when the shell thickness (*t*) varies from 100 to 5000 nm. The microbubble is filled with high-index liquid (dimethyl sulfoxide, DMSO, *n* = 1.479) and packaged by a low-index polymer (MY133, *n* = 1.33). The TOCs of the DMSO and fused silica are −5.4 × 10^−4^/K and 1.2 × 10^−5^/K, respectively [34,35]. For simplicity, we assume that the sensitivity is proportional to *κ_c_* and *κ_s_*. The radius of the microbubble is fixed to 100 μm. As shown in Figure 1a, the *S_T_* decreases monotonically with an increase in the shell thickness for the *p* = 1 mode. When the shell of the microbubble is thick (*t* = 3 μm, gray shadow), the majority of the electromagnetic field is confined within the shell, and only the evanescent field is tunneled to the interior environment (Figure 1d). Thus, the *S*_T_ is low (the black line in the Figure 1a). Conversely, when the shell of the microbubble is thin (*t* = 1 μm, gray shadow), the majority of the electromagnetic field is confined within the core (Figure 1d). However, this situation does not apply for the higher order radial modes (e.g., *p* = 2, 3). The *S*_T_ and *κ*_c_ for the *p* = 2 and *p* = 3 modes both oscillate as a function of *t*, which can be seen from Figure 1b,c. Thus, they are more complicated than the *p* = 1 case. It is worth noting that higher radial modes occupy more space than the first-order mode. When the shell thickness becomes thinner, the *p* = 2 and *p* = 3 modes still have a significant part of electromagnetic field in the microbubble wall. In this situation, the wall mode and the liquid core mode interact to couple, forming bonding and anti-bonding modes [33,36,37].

**Figure 2:**
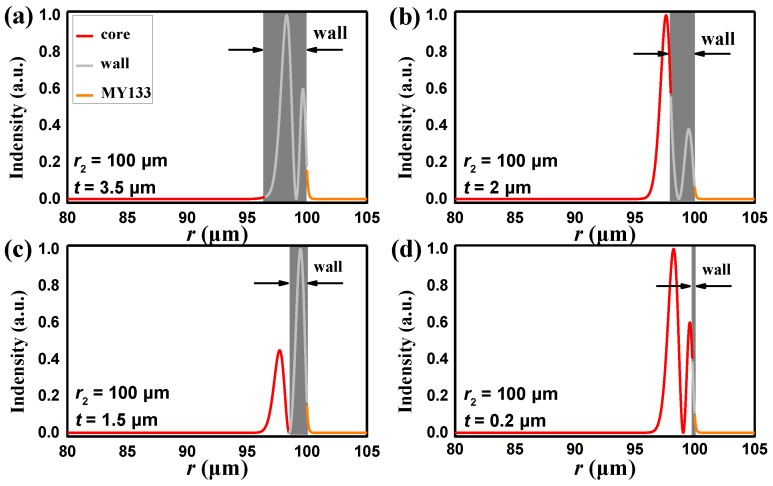
The whispering gallery mode (WGM) electromagnetic field distribution of the *p* = 2 mode for various shell thickness (gray shadow). (**a**) 3.5 μm. (**b**) 2 μm. (**c**) 1.5 μm. (**d**) 0.2 μm.

**Figure 3:**
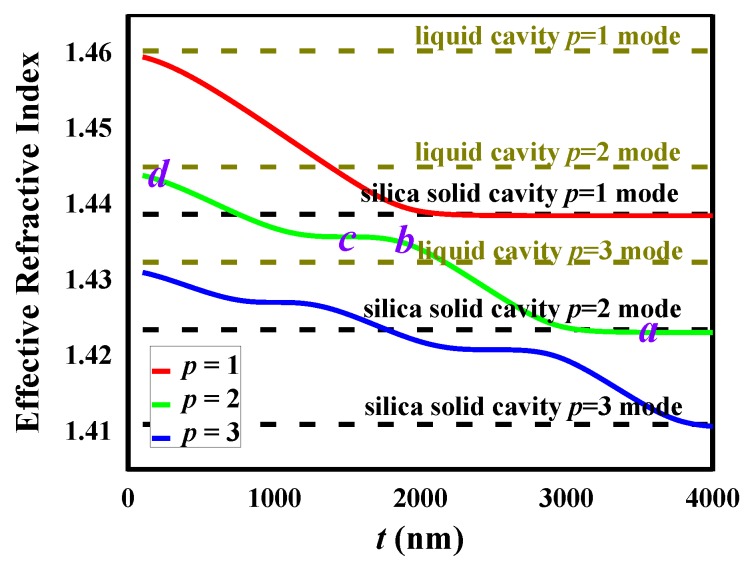
The effective refractive index as a function of the shell thickness for the first three radial WGM. The points (a, b, c, d) in the figure corresponds to Figure 2.

Figure 2 shows the electromagnetic field distribution of the *p* = 2 mode for various shell thicknesses where a process of the bonding mode and anti-bonding mode can be easily described. In Figure 2a, the shell thickness is very thick (*t* = 3.5 μm, gray shadow), the WGM field is mainly localized in the microbubble wall, and it can be called the wall mode. As shown in Figure 2b,c, when the shell thickness decreases to 2 μm and 1.5 μm, respectively, the mode extended into the core and the mode coupling emerged. When the shell thickness is 0.2 μm or less, the mode almost entirely propagates in the liquid core (Figure 2d). The WGM transforms into pure so-called core mode. Figure 3 explains the situation of the oscillatory characteristics by effective refractive index. The points (a, b, c, d) (denoted in the Figure 3) are corresponding to the field distributions in Figure 2a–d. For *t* values larger than 3 μm, the effective refractive index of *p* = 2 mode is the same as that of the WGM in a solid silica microsphere with the same size. The effective refractive index tends to that of the WGM in a liquid microsphere when the shell is thinner than 1 μm.

**Figure 4:**
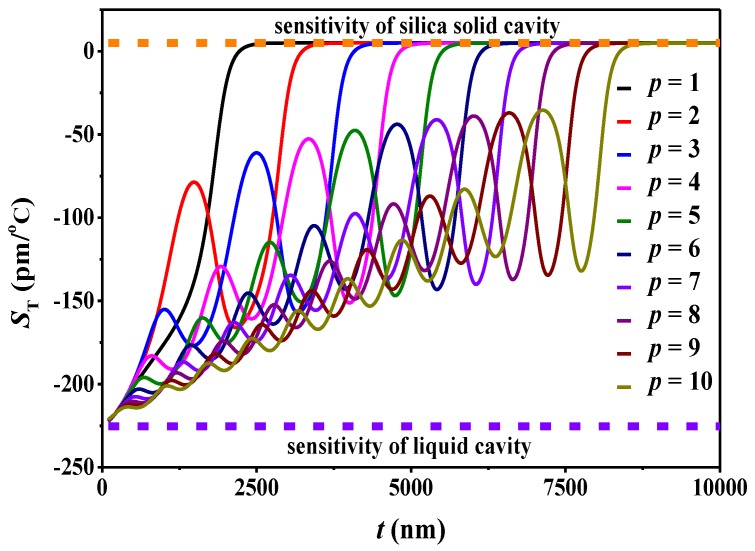
The sensitivity as a function of the shell thickness for the *p* = 1–10 WGM. The simulation parameters are the same as those in Figure 1.

The thermal sensitivity of more radial modes has also been analyzed. As shown in Figure 4, the thermal sensitivity oscillates multiple times for the higher order modes. When the microbubble wall is thick enough, all modes are confined within the shell, equivalent to a solid microsphere. At this time, all modes nearly reach the same sensitivity, which is the same as the sensitivity of solid silica microsphere [38,39]. The TOC of silica is positive, so the thermal sensitivity of solid silica microsphere is positive too. For example, the thermal sensitivity reaches 5 pm/°C for *p* = 1–10 modes when the shell thickness is larger than 10 μm (Figure 4). On the other hand, when the shell thickness approaches 0, all modes are confined within the liquid core. Now the microbubble cavity is equivalent to a droplet cavity, and the effective diameter is equivalent to the diameter of the liquid core. The thermal sensitivity in this extreme situation is the same as that of the microdroplet cavity with a thermal sensitivity of −225 pm/°C.

The research of “quasi-droplets” has been studies on the microbubble, which is filled with low-index liquid (*n* = 1.33) with a thin wall [4,30,31]. In the previous studies, most of the electromagnetic field distribution inside the liquid core should occur only in the case that the thickness is sufficiently thin; then, the microbubble becomes a so-called quasi-droplet cavity. From the simulation result (Figure 1), when the microbubble is filled with high-index liquid, the oscillatory distribution of the electromagnetic field will occur due to mode coupling. In this case, the quasi-droplet cavity with a high sensitivity can be achieved with a thick wall. A new quantified definition for a quasi-droplet is presented here. The regime where the percentage of the electromagnetic energy in the core is more than 50% is defined as the quasi-droplet regime (cyan shadow in Figure 1). In the quasi-droplet regime, the *S_T_* is more than 112.7 pm/°C (red broken line in Figure 1a). Thus, the sensitivity can be a good indicator of whether it is a quasi-droplet in our article. Due to the oscillatory distribution of the electromagnetic field, the quasi-droplet regime is discontinuous. For example, for *p* = 2 mode, the quasi-droplet regime is 1.78 μm < *t* < 2.7 μm and *t* < 1.1 μm (Figure 1b, cyan shadow). For *p* = 3 mode, the quasi-droplet regime is 2.8 μm < *t* < 3.7 μm and *t* < 2.1 μm (Figure 1c, cyan shadow). For a higher order mode, it can also realize the quasi-droplet region with a thicker shell, which is different from previous studies [4,30]. To the best of our knowledge, it is the first quantified definition of a quasi-droplet without requiring a thin wall in the microbubble, and it is important for the thick-wall microbubble to enhance the interaction between the WGM and the fluidic liquid.

## 3. Experimental Setups

**Figure 5:**
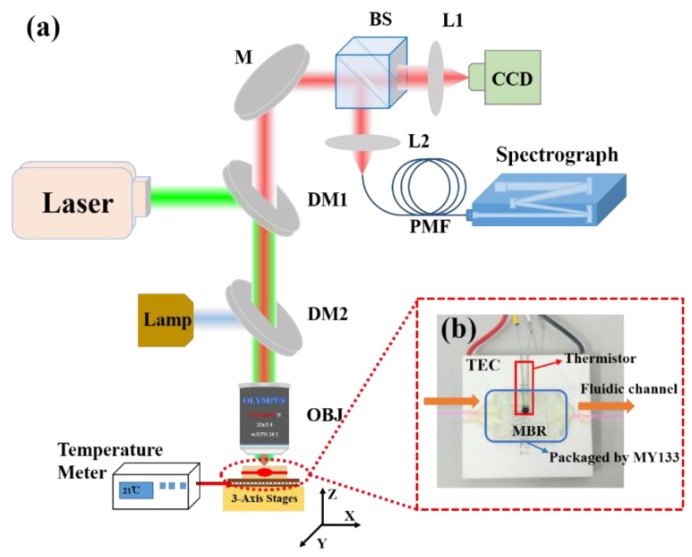
(**a**) The experimental setup schematic diagram of the WGM thermal sensor. OBJ: objective; M: mirror; L: lens; BS: beam splitter; DM: dichroic mirror; PMF: polarization maintaining fiber. (**b**) Optical image of the configuration of packaged MBR.

The detailed fabrication process of the microbubble can be found in previous work [40]. One end of the fused silica capillary is fused, and the other end is connected to a needle cylinder to allow air to be pressed into the tube. When the capillary is heated by the fusion splicer, the air is pushed into the tube at the same time. Due to the internal pressure, a microbubble is formed. Both of the ends of the capillary are connected to a Teflon tube to build a fluidic channel, enabling liquid to flow through the microbubble. A syringe micropump is used to transport and change fluid liquids. Figure 5a shows a schematic diagram of the experimental setup. The R6G/DMSO in the microbubble was pumped by a 532 nm ns-pulsed laser source (Ekspla NT340, repetition rate: 10 Hz, 5 ns pulses). A power meter (Newport model 1936-R) was used to measure the pulse power focused on the packaged microbubble. The excited and emitted lasers of the microbubble are focused and collected both in the free-space optics by an Olympus microscope objective (20×, Numerical Aperture = 0.4). Next, a beam splitter is employed (BS, 3:7) to divide the laser emission beam into two beams; one is sent to a CCD camera for imaging, and the other is finally coupled out via an optical fiber bundle which is directly connected into the monochromator (HORIBA iHR550, resolution: 0.1 nm).

As shown in Figure 5b, a thermoelectric cooler (TEC, 4 cm × 4 cm, Tianjin Zhongke Electric Thermal Co. Ltd., Thanjin, China) is used as the substrate. Two small glass slides are assembled on the TEC in order to obtain a region where the capillary could be placed. Both ends of the microbubble were glued to the glass substrate by using a UV curable epoxy. Next, the microbubble was packaged by the low refractive index UV glue (MY133, *n* = 1.33, MY POLYMERS Ltd., Ness Ziona, Israel) and solidified by a UV lamp for 5–10 min. The TEC and a thermistor (TH10K, Thorlabs Inc., Newton, NJ, USA) were connected to a temperature control system (TED4015, Thorlabs Inc., Newton, NJ, USA). The thermistor as a thermos probe was embedded in the polymer to monitor the sample temperature and generate feedback to the temperature control system. The TEC can keep the temperature stable by cooling or heating through feedback from the thermistor. The presented configuration exhibits three key advantages. Firstly, cured MY133 can prevent the surface of the microbubble from being contaminated or damaged. Secondly, the heat conduction was more uniform from the TEC to the microbubble as the MY133 can act a good thermal conductor. Finally, the robustness of the packaged structure (on chip) was further strengthened.

## 4. Measurement and Analysis

### 4.1. Lasing Characteristics and Stability Analysis in Packaged MBR

**Figure 6:**
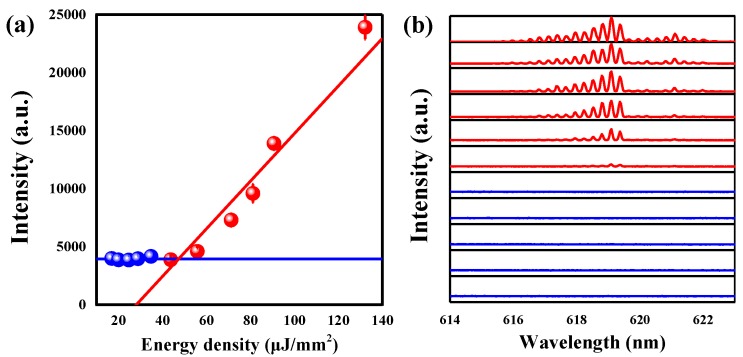
(**a**) Output laser intensity as a function of the energy density (light–light curve). (**b**) Evolution of the normalized output intensity as a function of wavelength and pump power.

In the experiments, the DMSO doped with Rhodamine 6G (R6G, 0.2 mM) is used and injected into the microbubble by the syringe micropump, which acts as both the gain materials and fluid liquids. A microbubble with a diameter of 200 μm and a thickness of 15 μm was packed by the MY133. Additionally, the use of MY133 also allowed the heat transfer channel to precisely control the cavity temperature. To verify the lasing behavior, a threshold measurement was taken. Figure 6 shows the peak intensity of the laser as a function of the energy density. A clear laser action is observed, and the curve shows that the lasing threshold is approximately 47.3 μJ/mm^2^. A plot of the evolution of the output powers as a function of both the pump power and the wavelength is shown in the Figure 6b. At low pump power, the intensity of the spectrum signal is low. When the pump power increases, the obvious narrow peaks win out from the spectrum. The lasing threshold is relatively higher compared to other lasing microresonators [4,41]. The reasons for this are as follows: (i) in our structure, the microbubble is packaged by polymer (MY133). Polymer absorption and surface scattering will cause the required pumping power density to become higher. (ii) The fluorescence/lasing properties of dye and dye concentration can also affect the lasing threshold. (iii) The lasing threshold is inversely proportional to the *Q*-factor [42]. The encapsulation by MY133 may cause the *Q*-factor reduction in the microbubble. Considering the photobleaching of the dye, a shutter was set before the laser into the objective to control the exposure time, and the short collecting time must be adopted (less than 1 min).

**Figure 7:**
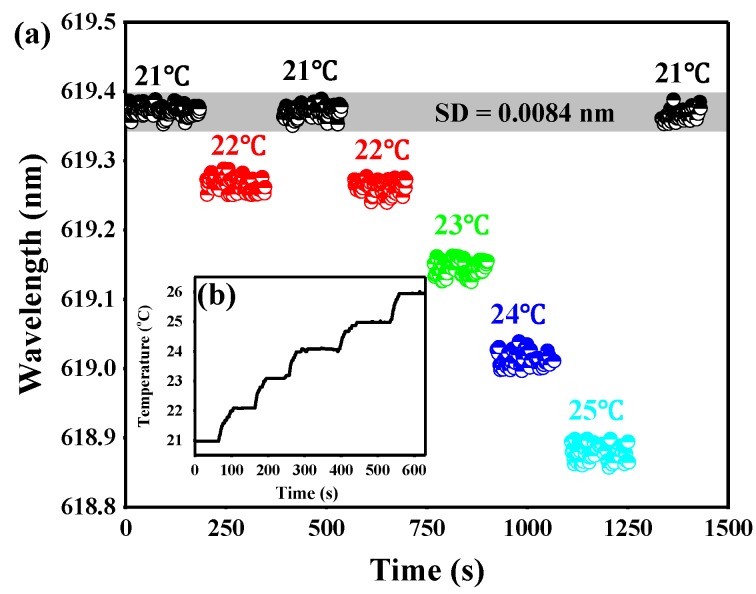
(**a**) The resonance wavelength plotted against time as the temperature was cycled. The temperature was set as 21 °C, 22 °C, 21 °C, 22 °C, 23 °C, 24 °C, 25 °C, and 21 °C. (**b**) The temperature as a function of time.

To evaluate stability and repeatability of the sensing system, we designed a series of temperature changes and recorded the data of the laser spectra. In this test, the temperature was set at 21 °C, 22 °C, 21 °C, 22 °C, 23 °C, 24 °C, 25 °C, and finally at 21 °C by a controlled heating stage. The results shown in Figure 7a indicate that the wavelength shift was identical to responses to the temperature change. The laser spectrum exhibits blue-shift when the temperature ranges from 21 °C to 25 °C. When the temperature cycles to 21 °C, the resonance wavelength also returns to the initial value with minute variations. As shown in Figure 7a, the standard deviation of wavelength shift is 0.0084 nm, which indicates that the fluctuation of temperature is 0.06 °C. Therefore, there is enough stability for us to record the resonance wavelength shifts. The temperature as a function of time is plotted in Figure 7b. We can see that the temperature was allowed to stabilize for 60 s before making a measurement. The detection range of the thermal sensor can be set from 19 to 55 °C, which is limited to the freezing point of DMSO and the heating range of TEC. Fortunately, the detection range can be extended to a wider range (such as 0–80 °C) by replacing the liquid material and TEC.

### 4.2. Packaged Quasi-Droplet MBR

**Figure 8:**
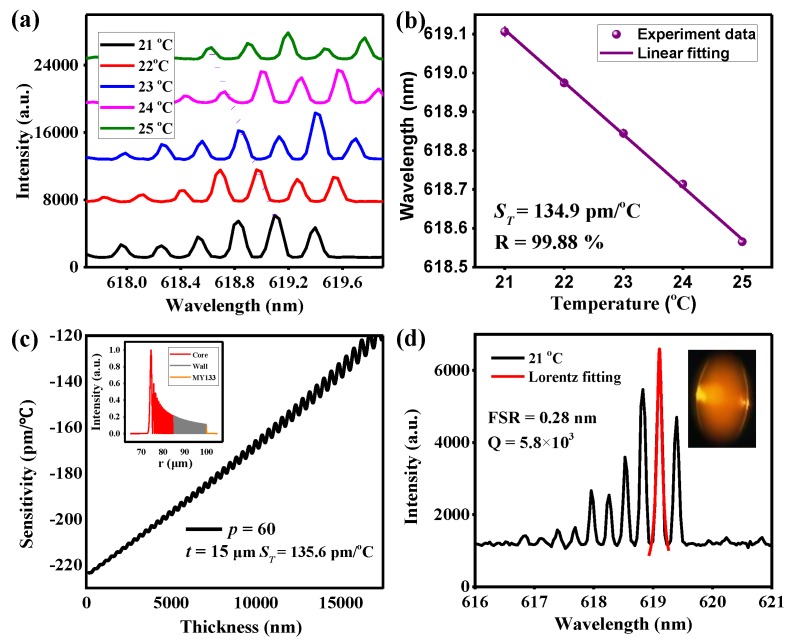
(**a**) The laser spectra of the WGM resonance at a temperature from 21 to 25 °C, with 1 °C increments. (**b**) The lasing resonance wavelength shifts as a linear function of the temperature. (**c**) The radial mode number was calculated to be 60. Inset, the electromagnetic field distribution of *p* = 60 in *t* = 15 μm. (**d**) The laser spectra of the WGM resonance at 21 °C. Inset, the CCD image of excited BMR.

Figure 8 shows the thermal sensing experimental results of packaged quasi-droplet MBR. When the temperature ranges from 21 to 25 °C, with the temperature increasing in 1 °C increments, the spectra exhibited a gradual blue-shift, as shown in Figure 8a. The resonant wavelengths change linearly with temperature, as shown in Figure 8b. The slope is −134.9 pm/°C, which is higher than −112.7 pm/°C (dividing line for the quasi-droplet regime). Figure 8c plots the calculated sensitivity distribution of radial *p* = 60 mode, and the inset shows the electromagnetic distribution with *t* = 15 μm. The calculated sensitivity of the *p* = 60 mode for *t* = 15 μm is −135.6 pm/°C, and more than 70.9% of energy is confined in the liquid core (inset in Figure 8c). The measured *S*_T_ matches very well with the calculated value of the quasi-droplet mode. As a result, high thermal sensitivity based on the packaged MBR operating at the quasi-droplet regime has been demonstrated even with a very thick but robust wall. The illustration in Figure 8d is a CCD image of excited MBR, and its laser spectrum is plotted in Figure 8d. The *Q*-factor (*Q* = *λ*/Δ*λ*, where Δ*λ* is the linewidth of the peak) and the free spectral range (FSR) of the MBR are 5.8 × 10^3^ and 0.28 nm. It should be noted that this is not a reliable accuracy measurement of *Q*-factor. The low effective *Q* may be caused by the following reasons: on the one hand, the linewidth of the lasing peak is limited to the resolution of the spectrometer, but on the other hand, the ability of light confinement in the microbubble may be weakened with packaging by MY133, which has a refractive index of *n* = 1.33, which is larger than that of air. The absorption loss induced by MY133 will also make the *Q* factor lower. The detection limit (defined as DL = Δ*λ*_min_/*S*) is ~0.74 °C for the MBR [43,44]. To improve the detection limit, one way is to take a higher resolution spectrometer, while the other is to increase the sensitivity of the sensor. According to theoretical analysis, to achieve high sensitivity, one should decrease the shell thickness. As an alternative approach, the material for both the microbubble and the liquid inside can be replaced by higher TOC material to increase the sensitivity.

### 4.3. Packaged Droplet WGM Resonator

**Figure 9:**
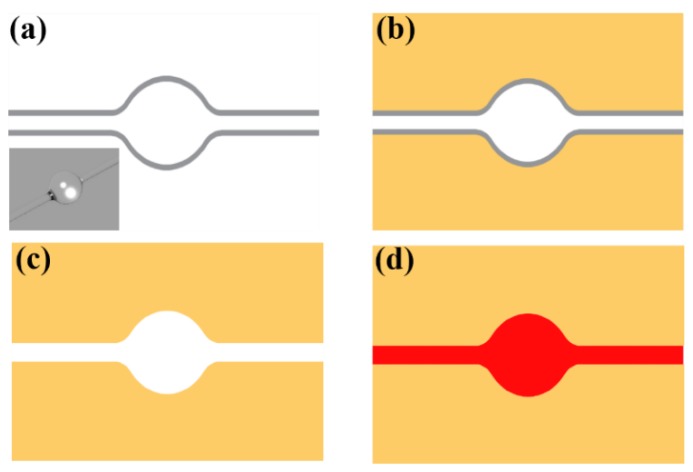
Schematic of the etching process: (**a**) A microbubble was fabricated by fuse-and-blow method. (**b**) The microbubble was packaged by MY133. (**c**) Etching the wall of the microbubble by HF. (**d**) The pure liquid cavity was packaged by MY133 and filled with DMSO (R6G).

As shown in Figure 1 and Figure 4, the sensitivity of the sensor increases as the wall thickness decreases. For the higher sensitivity, we study the packaged microbubble without a wall. In further processing, we etch the wall of the microbubble with hydrofluoric acid (HF) and obtain an on-chip, all-liquid-droplet WGM resonator. After 4% HF flows through the microbubble for 4 h with a flow rate of 20 μL/min, the wall of the microbubble is fully removed. The evolutionary process is shown in Figure 9.

**Figure 10:**
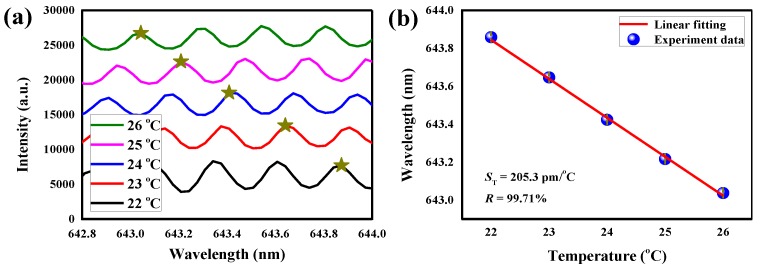
(**a**) The laser spectra of the WGM resonance at the temperature range from 22 to 26 °C. (**b**) The lasing resonance wavelength shifts as a linear function of the temperature.

The experimental results of packaged droplet WGM resonator are shown in Figure 10. As shown in Figure 10a, the WGM spectrum experiences blue-shift when the temperature ranges from 22 to 26 °C with 1 °C increments. Figure 10b shows the resonance wavelengths as a function of temperature. The ratio of the peak wavelength and the temperature is linear. The sensitivity of the packaged thermal sensor is −205.3 pm/°C. This value is slightly lower than the theoretical value (−225 pm/°C). One reason for this may be that the inner surface of the MY133 etched by HF is not very smooth, so the *Q*-factor and sensitivity decrease. Compared with some solid WGM thermal sensors, our packaged droplet WGM thermal sensor has higher sensitivity [45,46]. In addition, the microfluidic structure demonstrated in our optofluidic resonator structure makes the liquid delivery and replacement much easier. The sensitivity of the sensor can be further improved by replacing the liquid core with a higher TOC liquid material (such as heptane or hexane) [34]. For future biomedical applications, surface modifications can be conducted more easily with the polymer package for detecting bioactive molecules [47].

## 5. Conclusions

In conclusion, an effective method to realize the quasi-drop cavity with a thick wall in packaged MBR had been proposed and experimentally demonstrated. The optical properties of packaged MBR filled with high-index liquid has been simulated and analyzed using the Mie model theory. The oscillation distribution of the electromagnetic field resulting from the mode coupling makes a highly sensitive quasi-drop cavity with a thick wall possible. Additionally, the quantified definition of a quasi-droplet in a microbubble is presented here for the first time. A quasi-droplet thermal sensor with a sensitivity of 134.9 pm/°C (at temperatures ranging from 21 to 25 °C) has been experimentally achieved in a thick-wall (15 μm) microbubble. Furthermore, a packaged droplet WGM resonator is acquired by etching the microbubble shell with HF. The thermal sensitivity is as high as 205.3 pm/°C at temperatures ranging from 22 to 26 °C. In future work, a higher sensitivity and wider range of detection (0–80 °C, for example) can be obtained by changing the liquid material through the microflow channel. The fabricated thermal sensor is superior due to its ultrahigh sensitivity and robust properties, which have significant potential in environmental monitoring, biomedical applications, and photothermic devices.
